# Automated Assessment of Green Infrastructure Using E-nose, Integrated Visible-Thermal Cameras and Computer Vision Algorithms

**DOI:** 10.3390/s25226812

**Published:** 2025-11-07

**Authors:** Areej Shahid, Sigfredo Fuentes, Claudia Gonzalez Viejo, Bryce Widdicombe, Ranjith R. Unnithan

**Affiliations:** 1 Department of Electrical and Electronic Engineering, University of Melbourne, Parkville, Melbourne, VIC 3010, Australia; areejs@student.unimelb.edu.au (A.S.); bryce.widdicombe@unimelb.edu.au (B.W.); 2 Digital Agriculture, Food and Wine Group, School of Agriculture and Food, Faculty of Veterinary and Agricultural Sciences, University of Melbourne, Parkville, Melbourne, VIC 3010, Australia; sigfredo.fuentes@unimelb.edu.au (S.F.); cgonzalez2@unimelb.edu.au (C.G.V.)

**Keywords:** electronic nose, urban green infrastructure, vegetation indices, air pollution, predictive modelling

## Abstract

The parameterization of vegetation indices (VIs) is crucial for sustainable irrigation and horticulture management, specifically for urban green infrastructure (GI) management. However, the constraints of roadside traffic, motor and industrially related pollution, and potential public vandalism compromise the efficacy of conventional in situ monitoring systems. The shortcomings of prevalent satellites, UAVs, and manual/automated sensor measurements and monitoring systems have already been reviewed. This research proposes a novel urban GI monitoring system based on an integration of gas exchange and various VIs obtained from computer vision algorithms applied to data acquired from three novel sources: (1) Integrated gas sensor data using nine different volatile organic compounds using an electronic nose (E-nose), designed on a PCB for stable performance under variable environmental conditions; (2) Plant growth parameters including effective leaf area index (LAIe), infrared index (Ig), canopy temperature depression (CTD) and tree water stress index (TWSI); (3) Meteorological data for all measurement campaigns based on wind velocity, air temperature, rainfall, air pressure, and air humidity conditions. To account for spatial and temporal data acquisition variability, the integrated cameras and the E-nose were mounted on a vehicle roof to acquire information from 172 Elm trees planted across the Royal Parade, Melbourne. Results showed strong correlations among air contaminants, ambient conditions, and plant growth status, which can be modelled and optimized for better smart irrigation and environmental monitoring based on real-time data.

## 1. Introduction

The urban green infrastructure (GI) has been severely affected by the industry’s advances and rising chemical wastes in the environment [1]. Therefore, preserving the green belt in streets, parks, gardens, residential areas, and greenhouses has become increasingly challenging. Urban nature and GI, a source of biodiversity, regulate environmental temperature [2] and remove many air toxins [3] by producing fresh oxygen and carbon dioxide sequestration via photosynthesis [4]. The maintenance of GI aids in combating heatwaves [5], air pollution [6], global warming, floods, and landslides. However, with the rapid expansion of industrial developments, inflammable, odourless, and colourless pollutants escape into the environment, exposing natural life to contaminants. Several studies have reported air quality monitoring systems, such as the development of electronic-nose (E-nose) devices [7,8,9] and associated machine learning (ML) and artificial intelligence (AI) algorithms [10,11,12,13]. An E-nose mimics the human olfactory system using an array of detectable gas sensors. The air contaminants that can be considered threats to GI and human health are primarily volatile organic compounds (VOCs) that can be detected, identified, and quantified using recent advances in nano-scale gas sensing [14,15]. Metal-oxide semiconductors (MOS), such as zinc oxide (ZnO) [16], tin oxide (SnO_2_) [17,18], tungsten oxide (WO_3_) [19,20], indium oxide (In_2_O_3_) [21,22], manganese oxide (Mn_3_O_4_) [23,24] have been reported to have exceptional sensitivity for gases including hydrogen (H_2_), nitrogen oxide (NO_2_), formaldehyde (HCHO), ammonia (NH_3_), chlorine (Cl_2_), hydrogen sulphide (H_2_S), methane (CH_4_), acetone, ethanol, among others [25]. Similarly, field-effect transistors (FETs) have emerged as a promising technology for room-temperature gas sensing with higher sensitivity and stability, easy fabrication process, and fast response-recovery timeouts [26]. Despite having many advantages, such as multi-gas sensing, rapid response, and high sensitivity, these technologies have been recently developed, uncommercialized, and are currently expensive to deploy. Nonetheless, the SnO2-based gas sensors have a wide range of detectable gases [17,18], with a sensitive spectrum well within the target concentrations to monitor the environmental safety and predict its impact on the GI. Specifically, the low-cost, fast, and portable SnO_2_-based commercialized sensors with an established history of remote monitoring of predefined gases regarding method detection limits (MDL) seem arguably important for real-time urban pollution control. The MOS surfaces have a gas-sensing tendency to a diverse set of volatile species due to the surface film oxidation ability.

There have been numerous studies that have investigated the conditions of urban trees through data acquisition of parameters, such as soil moisture [27], nitrogen levels [28,29,30], light intensity [31], pH levels [32], humidity, and temperature [33]. The manual inspection of these attributes is inefficient and time-consuming. The automation of the inspection process has been achieved by deploying the monitoring setup by trained personnel for better spatial and temporal data resolution. These wireless sensor networks and IoT systems require routine supervision and debugging for quality assurance, which is costly and limited in scope. To overcome these limitations, other approaches have been proposed using satellites [34,35] and unmanned aerial vehicles (UAVs) [36,37]. The satellite images from Landsat 7–8 provide low-resolution images, while WorldView 3 and 4 offer high resolution at high cost. The satellites overpass an identified location every 12 days, limiting the data sampling rate. For higher spatial resolution, Jinru et al. [38] investigated and validated vegetation indices (VIs) acquired through UAVs, establishing that combining visible and NIR bands improves VI detection. Andrew et al. [39] discussed urban land cover classification using aerial photos and LiDAR datasets. However, the implementation of UAVs for high spatial and temporal resolution complicates the operation through high investment costs, dependence on expert personnel, and short flight time [40]. Also, the ability and civil aerial regulations in many countries to fly multispectral camera UAVs in urban zones are limited. Commercial platforms and databases, such as Landchecker [41], Nearmap [42], and OpenAerialMap [43], provide high-resolution visible images that can be used to supervise plant growth.

The application of computer vision algorithms for monitoring plant physiological status and growth has been previously reported to enhance urban GI’s predictive and descriptive analysis [44,45]. For instance, the comprehensive description of leaf area index calculation using computer vision algorithms [46] and its extension for urban GI surveillance [47] have opened new avenues in visible/thermal spectroscopy and computer vision for smart agriculture purposes.

This study proposes a novel mobile monitoring system integrating computer vision approaches to obtain critical vegetation indices, a low-cost E-nose, and meteorological information. This study contributes significantly to linking real-time plant and irrigation status with air pollutants (VOCs) and traffic situations in urban GI to study a correlation between environmental pollutants and GI parameters. With the growing inclination for plantations across urban roadside to maintain the GI, the hazards of environmental pollution and potential drought for these plants are also inevitable. It is proposed an efficient, cheap, and innovative solution for automated monitoring of urban GI using commercially available sensors and cameras to manage the impacts of air pollution and inadequate irrigation management over the urban GIs. Based on the statistical analysis and predictive modelling in this study, this paper proposes utilizing the acquired and processed data to monitor various plant and environmental statuses. These models can be readily implemented on any surveillance vehicle or public transport for continuous and repetitive measurements and calculations without additional infrastructure costs.

## 2. Materials and Methods

### 2.1. Urban Infrastructure Test Site

The test site selected for monitoring the urban GI is located in the heart of Greater Melbourne, Australia. The measurement site, Royal Parade Avenue (Parkville suburb), consists of 172 Elm trees (*Ulmus* spp.), both ways, starting from Grattan Street (Location A: −37°48′2.27″ S; 144°57′26.27″ E; 33 m.a.s.l) to Park Street (Location B: −37°46′41.45″ S; 144°57′36.56″ E; 46 m.a.s.l). These trees were planted in 1900 and 1997 along with the partitions of access roads and main roads on both sides, creating 2.52 km long, four parallel nature strips mirroring the public transport’s Tram Route 19 lane. The City of Melbourne council irrigates and maintains these trees as a part of its Urban Forestry Strategy, which is currently responsible for 70,000 trees.

### 2.2. Weather Data from the Trial Site

The weather data accessed from the Australian Bureau of Meteorology [48] were taken at the Melbourne Olympic Park meteorological station (Station No. 086338), which is 3.4 km from Royal Parade. The proximity of the BoM station to the Royal Parade ensures the precise environmental information and validity of the procedure. The acquired variables included minimum temperature (T_min_), maximum temperature (T_max_), rainfall (R), direction of wind (W.D), speed and time of maximum wind (W.S and W.T_MAX_), 9 a.m. Temperature (T_9A.M._), 9 a.m. relative humidity (R.H._9A.M._), 9 a.m. wind direction (W.D_9A.M._), 9 a.m. wind speed (W.S_9A.M._, km h^−1^), 9 a.m. MSL pressure (MSL_9A.M._, hPa), 3 p.m. Temperature (T_3P.M._), 3 p.m. relative humidity (R.H._3P.M._), 3 p.m. wind direction (W.D_3P.M._), 3 p.m. wind speed (W.S_3P.M._, km h^−1^) and 3 p.m. mean sea-level (MSL_3P.M._) pressure (hPa).

### 2.3. Electronic-Nose

For air monitoring based on volatile organic compounds (VOCs), a multiple-sensor array was established to have a better pattern of gases interacting within the air monitoring. Nine different types of commercial SnO_2_ gas sensors were used to detect VOCs present in the measurement site. Specifically, the modules developed by Henan Hanwei Electronics Co., Ltd., Zhengzhou, Henan, China, namely MQ-3, MQ-4, MQ-7, MQ-8, MQ-135, MQ-136, MQ-137, MQ-138, and MG811, were used due to their high sensitivity and selectivity towards hydrocarbons. Although these sensors show cross-sensitivity traits, MQ-4 has higher responsivity for CH_4,_ whereas MQ-7 is more receptive to CO, with no sensitivity towards pollen or P.M. Table 1 shows the target gases, associated sensor modules, detectable chemicals, and method detection and quantitation limits. Since the MOS sensor response is affected by environmental changes, an auxiliary temperature and humidity device, AM2320 (Guangzhou Aosong Electronics, Guangzhou, China), was also described to ensure that the sensors are operated within specified temperature and humidity limits. Whilst gas concentration mapping is not required, the E-nose was calibrated for baseline readings prior to each measurement.

**Figure 1:**
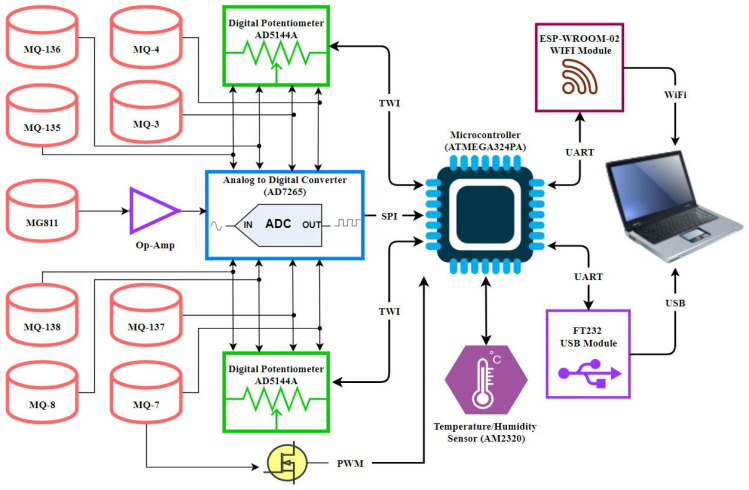
Block-level diagram of E-nose data flow starting from sensors (peripheral nodes), transmitting and processing through the microcontroller circuit to the local computer (controller node).

To ensure a stable operation, the printed circuit board (PCB) was designed with on-chip signal conditioning, microcontroller integration, and noise-filtering circuits. The E-nose setup assimilates a controller-peripheral architecture whereby the data acquisition components, i.e., sensors, constitute the peripherals and commands are executed via an onboard controller, i.e., a microcontroller. Signal conditioning and driver electronics are incorporated for each sensor. A microcontroller acquires sensor data via an analogue to digital converter (ADC) and processes it using firmware developed on Microchip Studio for AVR^®^ and SAM Devices. A graphical user interface (GUI) developed using the Python programming language version 3.11 (Python Software Foundation, Wilmington, Delaware, United States) captures the data at a user-defined sampling rate through a universal serial bus (USB) or Wi-Fi module. This data is logged in comma-separated value (.csv) files and plotted on the GUI in real-time on the user’s computer or laptop. The schematic illustration of the data flow inside an E-nose is shown in Figure 1.

The printed circuit board (PCB) circuit design was performed in ALTIUM Designer version 21.0.9 (Build 235). The four-layered E-nose PCB is a polygon shape measuring approximately 80 mm by 80 mm, strategically designed to have all gas sensors integrated on one side of the board and a control circuit on the other. This E-nose is the updated version of our previously published model [49] to enable multiple agricultural applications. The E-nose was fixed on the moving vehicle on a suction mount with sensors exposed to the outer environment and was powered through a USB cable.

### 2.4. Visible and Thermal Infrared Integrated Cameras

The FLIR Duo Pro R camera system (69 mm × 87 mm × 82 mm; FLIR Systems, Wilsonville, OR, USA), which is an integrated visible RGB video and thermal infrared camera, was also used. The pixel array of the visible camera is 4000 × 3000. This camera comprises a magnetic GPS antenna port, HDMI output, accessory port, USB-C port, two microSD card slots, a record button, and two lens barrels, one for each visible and IR camera. A bench cable connected to the camera via the accessory port was used to supply power through an XT60 connector (Jaycar Electronics, Rhodes, NSW, Australia). This cable has an integrated temperature/humidity sensor, AM2302 (Guangzhou Aosong Electronics Co., Ltd., Huangpu District, Guangzhou, China). The camera was mounted on top of the vehicle, oriented upwards (facing the sky) through a 1/4” -20 suction cup mount, which minimized the bounces while driving the car.

The camera was also connected to the FLIR UAS App 2.2.1 (FLIR Systems, Wilsonville, OR, USA) for iOS via Bluetooth to configure camera settings, initiate/stop recording (with an audible beep), and install any updates. The camera also had an integrated magnetic GPS tracker that logs the positional coordinates for each video frame. Other radiometric data included sky condition and object emissivity and were sampled at the rate of 1 Hz (every second). The instantaneous record of GPS coordinates and timestamps with each video frame (at a rate of 30 fps) plays a crucial role in positional targeting of data for all studied parameters.

The microSD cards (64 GB) were used to store raw thermal video files in 14-bit Tagged Image File Format (TIFF) on SD Card 1 and the remaining data on SD Card 2. The first frame of each .tiff sequence contained the radiometric data. The visible videos were saved in 1080p formats as QuickTime multimedia file format (.MOV) files. There is a SubRip Subtitle (.SRT) file of the same name with each video file containing the metadata of the GPS coordinates, altitude, time, and date. The camera was remotely controlled through the FLIR UAS App during all measurement campaigns, and data were recorded on local microSD cards

### 2.5. Measurement Setup and Data Acquisition

**Figure 2:**
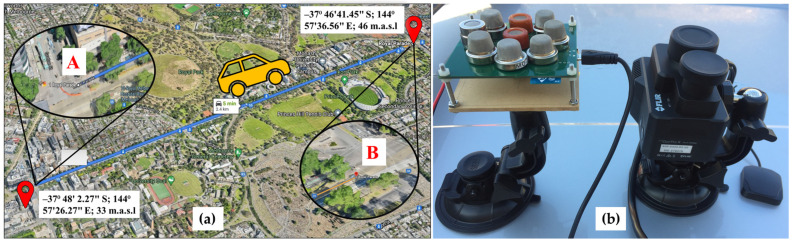
(**a**) Testing site at Royal Parade consisting of a green belt of Elm trees from location A at Grattan Street (−37°48′2.27″ S; 144°57′26.27″ E; 33 m.a.s.l.) to location B on the Park Street (−37°46′41.45″ S; 144°57′36.56″ E; 46 m.a.s.l.) and in reverse direction and (**b**) Measurement setup consisting of E-nose and integrated visible-thermal camera with magnetic GPS tracker mounted on the vehicle.

**Figure 3:**
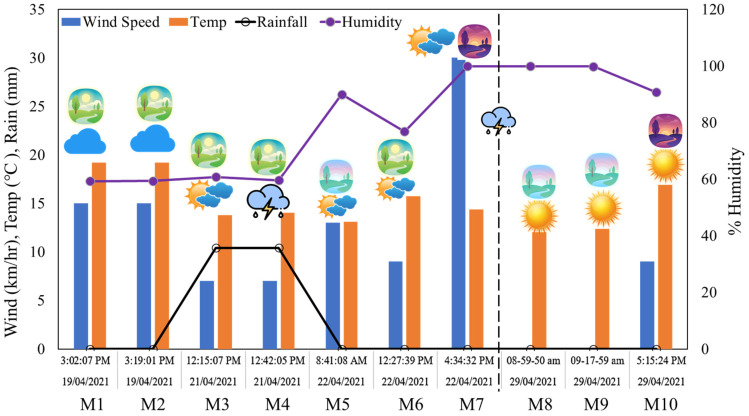
Data acquisition (10 measurement campaigns M1–M10) during April 2021 with date and time, day slot (morning, afternoon, or evening), and corresponding weather conditions in terms of wind speed, temperature, rainfall, and humidity.

The E-nose and FLIR camera were mounted with all the accessories on top of the car with a suction gimbal for motion stability and powered through a 12 V lighter socket connected via the front left car window (Figure 2b). Real-time data from the E-nose was visualized on a laptop GUI, and the camera was operated via Bluetooth connection with an iPhone 6 (Apple, Cupertino, CA, USA). Each set of measurement campaigns consisted of distance traversal of a total of 5.04 km (2.52 km one way) and acquired atmospheric sensory data, visible and thermal imagery data, and radiometric parameters. Data acquisition was performed on three contrasting weather days to ensure adaptability and inclusion: cloudy, partly cloudy, and clear/sunny. Also, to incorporate the atmospheric fluctuations due to various timings and conditions of the day, the trials were repeated in the morning, afternoon, and evening. This practice generated a versatile dataset with a wide range of spatial and temporal sample points. Specifically, the monitoring was performed in April 2021 on the days of the 19th (cloudy: M1, M2), 21st (partly cloudy: M3, M4), 22nd (partly cloudy/cloudy: M5–M7), and 30th (clear/sunny: M8–M10). Figure 3 lists the ten measurement campaign dates, times, day slots, weather conditions, and labels from M1 to M10. Each measurement session (M1 to M10) along the 5.04 km site was completed in 12–15 min (~5.6 m/s), whereas the E-nose sampling rate was 1 Hz, and that of the FLIR Camera was 30 frames per second. This temporal resolution was sufficient to capture diurnal variability across all monitored tree canopies.

### 2.6. Data Processing and Computer Vision Algorithms

The visible videos and thermal images were analyzed using customized codes developed and updated in MATLAB^®^ R2024b (MathWorks Inc., Natick, MA, USA) and Python. The visible videos were preprocessed by extracting the histogram curves (red, blue, and green) from the frames and thresholding the blue histogram. Two peaks were detected in all histograms corresponding to the tree canopy and background sky. The lowest spot between the two peaks of the blue histogram was manually selected for binarization. The images were then segmented for gap analysis into 5 × 5 sub-images. The criterion for a large gap (lg) was set to 75% sky for each sub-image as detailed in Fuentes et al. [46,47]. This data was used to calculate the effective leaf area index (LAIe, Equation (3)) along with other canopy growth parameters. An example of a processed tree canopy image is demonstrated in Fuentes et al. [47].

The leaf area index (LAI), the one-sided green leaf area per unit ground surface area, is an adimensional quantity that can be calculated using Beer’s law as:

(1) $$LAI=-f_{c}\frac{\ln\emptyset }{k}$$

where *k* is the coefficient of light extinction with a value of 0.5, *f_c_* is the canopy crown cover, and ∅ the crown porosity. In a non-random canopy distribution, the clumping index (Ω) at the zenith is a correction factor that can be found by:

(2) $$Ω\left(0\right)=\frac{\left(1-\emptyset \right)\ln\left(1-f_{f}\right)}{\ln\left(\emptyset \right)/f_{f}}$$

Using the above calculations, the effective LAI (LAIe) was calculated using:

(3) $${LAI}_{e}=LAI*Ω(0)$$

The LAI quantifies the total one-sided leaf area per m^2^ of soil [50]. The large gap pixels, total pixels in all gaps, and total gap pixels in all images are considered to calculate the aforementioned parameters [46,47].

Another MATLAB customized code was employed to analyze the radiometric data per pixel obtained from the thermal images. The GPS data associated with each frame assisted in mapping the studied variables to identify tree locations and stitch all parameters at the per-tree level using geo-location. These images were used to process the pixel matrix to select leaf material. This can be performed by selective filtering temperatures lower than 0+ °C that correspond to the tree canopy. The segmented image can be used to extract the canopy temperature (T_canopy_). Using the preprocessed data, we used an established computer vision technique proposed by Fuentes et al. [46,47] for the estimation of the tree water stress indicator (TWSI) and infrared index (I_g_).

The thermal images per tree were used for the calculation of TWSI and Ig. The crop water stress index (CWSI), a strong indicator of water deficiency in plants, can be calculated through canopy temperature readings. The TWSI is a feature-scaling normalized derivative of CWSI, i.e., dynamic range translated to [0, 1]. The statistical temperature distribution discrimination gives a lead for two reference temperatures, T_wet_ and T_dry_. These are the two extremes of the temperatures found in a canopy and can be measured via infrared thermography. The procedure can be automatic or manual by covering the leaves with water (T_wet_) or moisture-locking/water repellent petroleum jelly (T_dry_). After measuring the actual canopy temperature (T_canopy_), the TWSI can be calculated using the following equation:

(4) $$\mathrm{TWSI}=\frac{T_{canopy}-T_{wet}}{T_{dry}-T_{wet}}$$

similarly to the water stress indices, the canopy temperature depression (CTD) can also depict the tree water status and water stress conditions of the tree canopy. The increased environmental demand signals the plants of the potential drought situation, which leads to stomatal closure. As the ongoing transpiration is responsible for the cooling of the plants, the cessation of this process increases the canopy temperature (T_canopy_). The CTD parameter can show the plant water status as per the following formula:

(5) $$CTD=T_{canopy}-T_{average}$$

where T_average_ is the ambient air temperature collected through the environmental sensors (E-nose) corresponding to all measurement campaigns. The formulas listed in Equations (1)–(5) were applied to automation algorithms in MATLAB that read videos at a frame rate of 30 fps and calculated all growth parameters for every incident tree canopy. The results from the computer vision algorithms and data processing are discussed in the next section.

Building on the foundational study by Fuentes et al. [46,47], this research further advances the field by integrating E-nose technology with visible/thermal imaging for enabling a multi-modal and integrated approach that incorporates environmental parameters and thoroughly investigates the correlation with GI parameters.

### 2.7. Data Synchronization

For this study, GPS coordinates of the monitored Elm trees were obtained via Google Earth Pro (Mountain View, CA, USA). These tree positions were assigned an ID number and served as reference points for automated data extraction of the Tree Water Stress Index (TWSI), Infrared Index (Ig), Canopy Temperature Depression (CTD), and Leaf Area Index (LAI) based on previously described methodologies in Equations (1)–(5). Automated extraction involved identifying the nearest recorded coordinates from the integrated camera to each anchored GPS point. Linear interpolation is used to log GPS points (integrated timestamps) at a 10 Hz rate. Based on the trees’ ID and list of their GPS coordinates, a series of geofenced regions was created to minimize overlap between adjacent regions. The camera entrance and exit timestamps were calculated for each geofenced region, confined to a tree ID. An average of each parameter, including gas sensor values from the E-nose, was utilized for this tree ID, ensuring that timestamps and tree locations are aligned.

### 2.8. Statistical Analysis

The data from the E-nose was organized separately for each trip (M1−M10), consisting of sensor values across the three GPS coordinates and a timestamp. The mean values were extracted for each gas sensor corresponding to all measurement campaigns. The data was plotted in stacked bar charts to compare and analyze the temporal variations in measured mean gas sensor values. The relationship between gas emission signals, GI parameters, and environmental factors (including pressure and wind speed) was analyzed to evaluate atmospheric interference effects. The principal component analysis (PCA) was performed on E-nose data, vegetation indices, and weather data collectively for cloudy and sunny days. Using Pearson’s correlation coefficient (R), which measures the strength of a linear association between variables, a lower triangular correlation matrix was also developed for all these parameters, highlighting significant values. These plots were generated in OriginPro 2024b (OriginLab Corporation, Northampton, MA, USA).

### 2.9. Machine Learning

**Figure 4:**
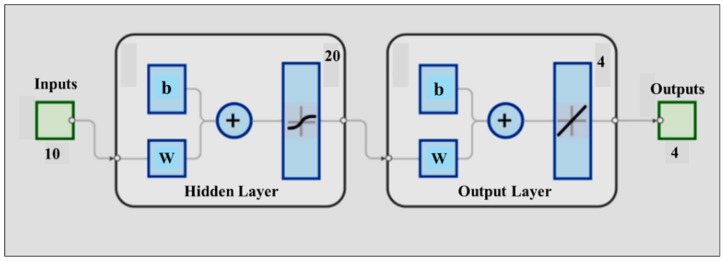
Schematic of a two-layer feedforward neural network with 10 input neurons and 20 hidden neurons (using sigmoid transfer functions) to fit regression models for four targets/outputs from a linear output layer.

The physiological parameters of the tree canopies (TWSI, Ig, CTD, LAI, and LAIe) that have been calculated using costly equipment (FLIR Duo Camera) and computer vision algorithms have predictability potential using the cost-effective equipment (E-nose) and free real-time BoM data. Therefore, an artificial neural network (ANN) regression model was developed using MATLAB 2024b (MathWorks, Inc., Natick, MA, USA) through the “*nftool*”. This ANN was designed to take sensory data with ten inputs (MQ-3, MQ-4, MQ-8, MQ-135, MQ-136, MQ-137, MQ-138, MG811, Temperature, Humidity) and estimate the four plant physiological parameters (TWSI, Ig, CTD, LAI) at the output layer. The hidden layer employed a sigmoid activation function with 20 neurons, chosen based on neuron trimming test (*n* = 5, 10, 15, and 20), and the training was implemented using the Levenberg–Marquardt algorithm. Before training, all variables were rescaled to the [0, 1] range. Sensory voltages were divided by 5, temperature by 50, humidity by 100, and LAI by 5 (the maximum LAI from the data was approximately 4.8). CTD values were scaled between −20 and +20, while TWSI and Ig were already normalized based on the formula listed in Equation (4). The data was split randomly into 70% for training, 15% for Testing, and 15% for validation by *nftool,* and the performance was assessed using the mean square error (MSE). Figure 4 below shows the schematic illustration of the feed-forward two-layer (hidden and output) neural network for estimating 4 outputs.

## 3. Results and Discussion

### 3.1. Weather Data from BoM

The weather information acquired from the BoM for all measurement campaign days included T_min_ (°C), T_max_ (°C), R (mm), W.D, W.S (km h^−1^), W.T_MAX_, T_9A.M._ (°C), R.H._9A.M._ (%), W.D_9A.M._, W.S_9A.M._ (km h^−1^), MSL_9A.M._ (hPa), T_3P.M._ (°C), R.H._3P.M._ (%), W.D_3P.M._, W.S_3P.M._ (km h^−1^) and MSL_3P.M._ pressure (hPa). The values of these parameters for the test measurement campaign days are listed in Table 2.

### 3.2. Air Monitoring via E-nose

**Figure 5:**
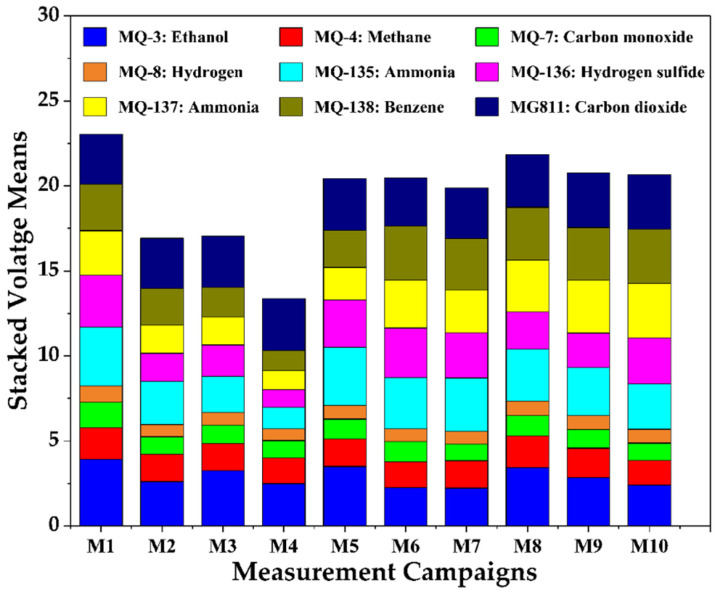
Diurnal mean stacked voltage values from gas sensors of E-nose on measurement campaigns conducted on cloudy and sunny days.

In addition to thermal radiometric data and plant physiology parameters, the mean gas sensor values (Figure 5) can generate a set of decisive conclusions on the state of urban GI. The data acquired during the first seven measurement campaigns (M1–M7) on cloudy and partly cloudy days were spread across 19, 21, and 22 April 2021. The E-nose’s stacked mean gas sensor values, as shown in Figure 5, were lower in the measurement campaigns on cloudy days than on sunny days. M1, the first measurement campaign, performed on a cloudy day, is an exception, with the highest voltage readings driven predominantly by ethanol (MQ-3) and benzene (MQ-138), which are attributed to traffic congestion and re-routing along the Royal Parade. This phenomenon is interestingly depicted in the contrast of E-nose values in M4, conducted in the early afternoon with minimal traffic, indicating lower pollution levels during these tests. These gases are also a result of vehicular emissions [51]. A significant rain event of 10.4 mm occurred on 21 April with high-speed winds of 30 km/h (Table 2) on 22 April afternoon, simultaneously leading to accelerated evapotranspiration and increased pollution content [52] as shown in M5−M7. Among these, the means for gas sensor values in M5 (08:40–08:50 a.m.) were higher because of the elevated concentrations of pollutants due to the peak traffic hours in the morning. However, the previous rain event (on 21 April) might cause comparatively lower mean gas sensor values (Figure 5) on cloudy days than on the following sunny days.

The last three measurement datasets (M8–M10) acquired on a clear/sunny day, 29th of April, show slightly higher overall cumulative voltage means, suggesting a moderate increase in pollution levels as the previous three days had minimal rain events (26th: 1.2 mm, 27th: 0.6 mm, 28th: 0.4 mm). This consistent presence of vehicular and industrial pollutants in the sensor readings suggests ongoing emission sources resulting in a variation in environmental pollution levels across the measurement campaigns, with ethanol and benzene, both common in vehicular emissions, being the most significant pollutants detected across the board. The elevated temperatures and higher traffic on sunny days have shown the much-expected rise in cumulative mean voltage values of all gas sensors. The data indicate that previous rain events, wind speeds, clear/cloudy sky, and traffic conditions are all key factors influencing pollution levels.

Air quality was generally worse on warmer and hot days due to gaseous emissions, particularly oxides of nitrogen and volatile organic compounds, from vehicles and industries undergoing complex reactions in sunlight’s catalytic activity. These chain reactions create photochemical smog, a toxic brown haze. Also, though growth parameters and weather data show significant variation across sunny and cloudy days, the E-nose can differentiate the diurnal gas data acquired at different timings across the 10 measurement campaigns.

### 3.3. Plant Water Status from Thermal IR Images

**Figure 6:**
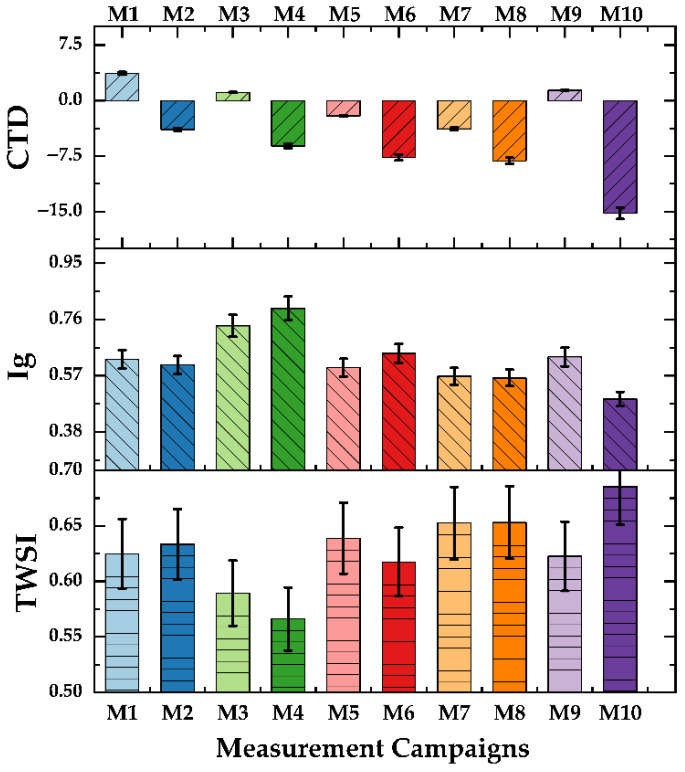
Bar charts of canopy temperature depression (CTD), infrared index (Ig), and tree water stress index (TWSI) for measurement campaigns performed on cloudy, partly cloudy, and sunny days obtained from meteorological data and computer vision algorithms.

Figure 6 illustrates the tree water stress and canopy growth parameters for cloudy, partly cloudy, and sunny days. The bar plots CTD, Ig, and TWSI plotted against the measurement campaign number (M1–M10) in Figure 6. For all weather conditions, higher TWSI values were associated with lower Ig values, indicating a negative relationship.

Also, CTD takes a negative value when transpiration is high. Conversely, Ig values decline as the CTD and TWSI rise, suggesting that higher stomatal conductance corresponds to increased heat stress. The variations in TWSI and LAIe also seem moderately related (positively), implying that soil moisture influences leaf area and transpiration rates. Also, in the later measurement campaigns conducted on sunny days (M8–M10), the increased water stress in high atmospheric demand on sunny days leads to stomatal closure of the plants and low transpiration. Thus, the T_canopy_ and, resultingly, the TWSI increase. Studies report that the TWSI is an indicator of plant water status and soil moisture content and; therefore, can be used as a reliable metric for automated irrigation systems [53,54]. Under specific growing conditions, large negative CTD values suggest that the stomata are fully open [55]. The attenuated magnitudes of CTD, on the other hand, are physiological indicators of decreased evapotranspiration, such as the values depicted in measurement campaigns M3 and M6. It can be inferred, using the bar charts in Figure 6, that as the windy days (19, 22 and 30 April) speed up the evapotranspiration (Wind Speed 9 am and 3 pm, Table 2), and sunny days induce high photosynthesis rate, the vectors of plant physiology, including Ig, CTD, TWSI and LAIe were more significant on sunny days as compared to cloudy days. Further insights into the interdependence of the studied parameters are evaluated through statistical analysis presented in the next sections.

### 3.4. Canopy Growth Monitoring Obtained via RGB Videos and Computer Vision Analysis

**Figure 7:**
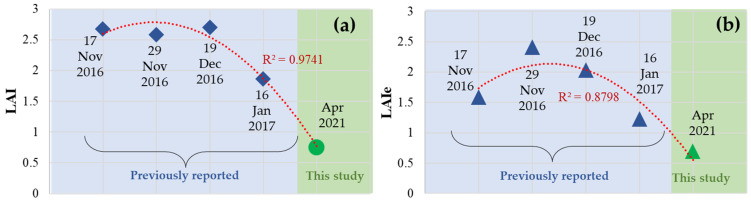
Data trendline for mean values of (**a**) LAI and (**b**) LAIe for previously reported [47] values in Nov 2016−Jan 2017 by Fuentes et al. and the current study (April 2021), depicting a predictable fit at the decline of LAI whilst approaching the senescence stage.

The LAIe parameter had slight magnitude variations on cloudy days, whereas they were observed to be consistent on the last measurement campaigns (sunny days). Also, the histogram plotted for canopy video frames using Fuentes’s algorithm [46] on sunny days showed a clear distinction between tree canopy and sky compared to those created for cloudy days. These histogram results were consistent with those reported by Fuentes et al. [46,47] and had two distinct peaks corresponding to the sky and canopy peaks on the left and right sides of the histogram. Table 3 enlists two examples of descriptive statistics, including minimum, maximum, mean, and standard deviation values for growth and water stress metrics acquired from measurement campaigns in April 2021. The average value of LAI and LAIe for all measurements acquired in this study was 0.748 and 0.697. There is a slight variation in minimum and maximum values for LAI/LAIe. However, a considerable decline in these parameters was observed with respect to the previously reported results by Fuentes et al. [47] for the months of Nov, Dec, and Jan (2016–2017). To validate the measurements, the trends were calculated in Figure 7 to observe a curvilinear decline in LAI/LAIe. Figure 7 demonstrates the decrease in the LAI/LAIe values for the measurement campaigns in April with respect to previously reported values for the months of Nov−Jan, attributed to the onset of autumn when trees had already started a senescence stage.

It is expected that weather conditions such as rain, wind, and sunlight will not have an immediate or direct effect on the LAIe of the plants. It is also previously reported in this study that the mean gas sensor values from the E-nose were generally higher on sunny days. While the relationship of LAI to organic pollutants and particulate matter contrasts, a few studies report that the dust retention capacity of some trees directly correlates to the LAI [52,56]. It can; therefore,, therefore, be rational to assume that the plants with better LAIe might be more capable of restraining the air pollutants and might also be effective purifiers. However, research supports that elevated CO_2_ levels might increase the maximum LAIe [57] primarily by improving photosynthetic efficiency, greater carbohydrate production, and delayed senescence. Therefore, it can be said that the LAIe is directly related to the CO_2_ gas over the span of a few years. With the prevalent rise in greenhouse gas emissions, E-nose technology is a promising and reliable measuring tool to incorporate with physiological parameters calculation to further investigate the relationship. Simultaneously, other toxic pollutants found in the environment can play a different role in the plants’ physiology and water status.

**Figure 8:**
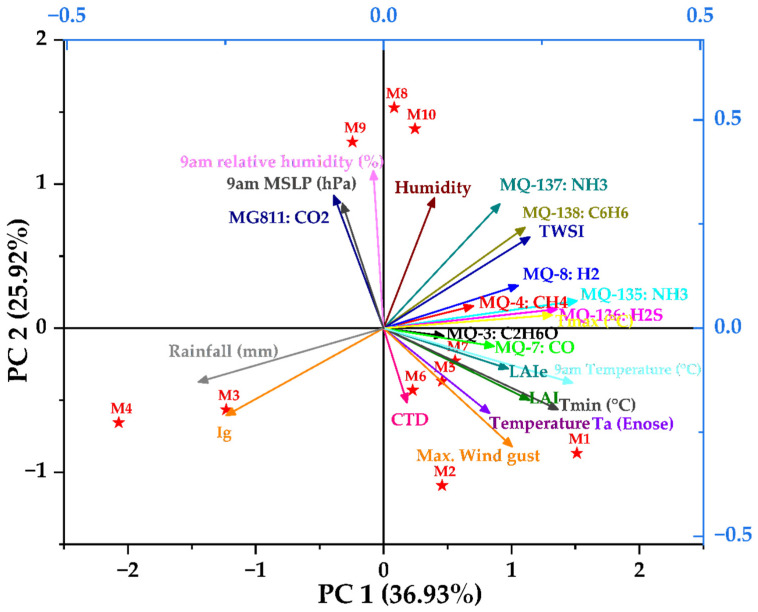
Principal component analysis of gas sensor data, leaf area index/effective (LAI/LAIe), canopy temperature depression (CTD), infrared index (Ig), tree water stress index (TWSI), minimum/maximum temperatures (Tmin/Tmax), and mean sea level pressures (MSLP) from measurement campaigns conducted during morning, afternoon and evening time in cloudy and sunny days.

**Figure 9:**
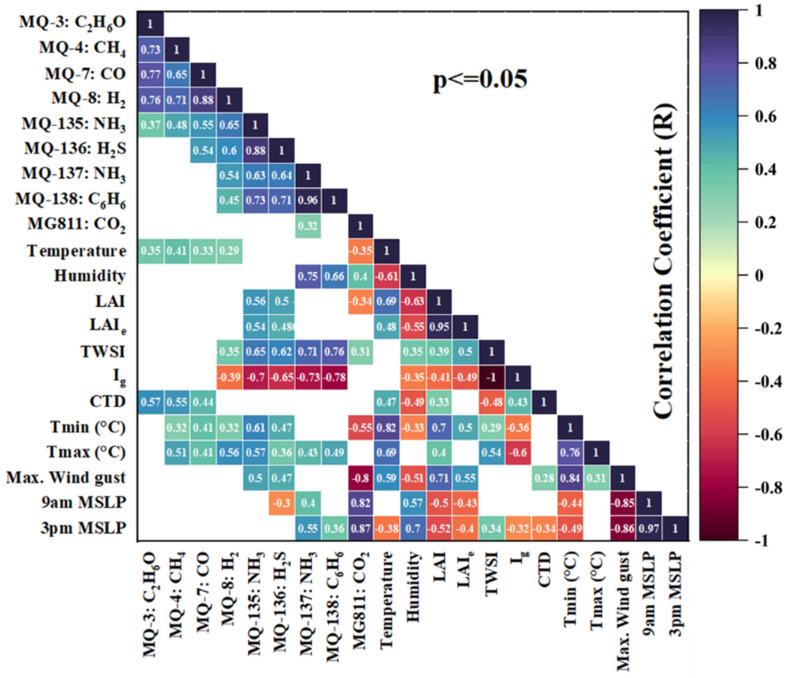
Correlation matrix of acquired data through the E-nose, integrated camera, and BoM website.

The PCA plot (Figure 8) and the correlation matrix (Figure 9) of mean gas sensor data, canopy growth parameters, water status, and meteorological data give insightful analysis to help automate and facilitate irrigation. Figure 8 shows the PCA using the data collected on cloudy days. It shows there was a total of 62.85% of data variability (PC1: 36.93%; PC2: 25.92%) using data from ten measurement campaigns. It can be observed that, based on the factor loadings (FL), the principal component one (PC1) was mainly represented by MQ-135:NH_3_ (FL = 0.305) and 9 am temperature (FL = 0.298) on the positive side of the axis, and Rainfall (FL = −0.292) and Ig (FL = −0.248) on the negative side. Principal component two (PC2) was represented by MG811:CO_2_ (FL = 0.318) and 9 am humidity (FL = 0.378) on the positive side, and Max Wind gust (FL = −0.284) and Ig (FL = −0.209) on the negative side of the axis. There were positive relationships between LAI, LAIe, Tmin, and all E-nose sensors (except MG811) and a negative relationship with Ig. TWSI has a strong (positive) correlation with MQ135, MQ136, MQ137, and MQ138. CTD has a positive correlation with MQ3, MQ4, and MQ7. E-nose sensor, Temperature, and Ig.

MG811 had a positive relationship with 9 am MSLP and 9 am relative humidity and an inverse relationship with the Max wind gust and LAI. TWSI has inverse relevance to Ig and CTD, but positive relevance to LAI, as also supported by the literature. There is a separation of the samples where they formed three groups: (M1, M2, M5, M6, M7) represented on positive PC1, (M3) represented majorly by Ig on negative PC1, and (M8, M9, M10) grouped together and associated with 9 am relative humidity and Humidity along positive PC2. The gas sensor parameters, canopy physiological indicators, and environmental factors all show an interesting pattern of correlation, which is also quantitatively verified in the next correlation coefficient matrix. This phenomenon opens avenues to apply machine learning to predict one class of variables using the acquired data from another category.

Figure 9 shows the correlation matrix that provides significant insights into the dynamics of air pollutants, tree physiological parameters, and environmental factors. Strong positive correlations (*p* ≤ 0.05) among sensors, such as ethanol (MQ-3) with methane (MQ-4: R = 0.73), carbon monoxide (MQ-7: R = 0.77), and hydrogen (MQ-8: R = 0.76), suggest that these gases often co-occur, likely emitted from shared sources such as vehicles or industrial processes. In contrast, benzene (MQ-138) shows weaker correlations (e.g., with MQ-8: R = 0.45), reflecting its distinct chemical behaviour and slower degradation in the atmosphere. All gas sensors are positively correlated except for a few exceptions, such as MQ-3:C_2_H_6_O (ethanol) and MQ-138: C_6_H_6_ (benzene). The R is valued at −0.11, which, although insignificant, implies a correlation. This phenomenon may indicate potential inhibitory effects of ethanol (C_2_H_6_O) on biodegradation properties for gasoline products such as benzene (C_6_H_6_), acting as a natural attenuator [58]. Hence, sensor readings are greatly impacted by environmental factors. Leaf Area Index (LAI) and effective LAI (LAIe) correlate negatively with CO_2_ levels (MG811: R = −0.34, R = −0.31), indicating that vegetation reduces CO_2_ concentrations through photosynthesis. However, LAI has limited effects on other gases, suggesting its primary role is in CO_2_ absorption rather than pollutant reduction. The weather conditions, such as wind speed, play a significant role in pollutant dispersion.

Maximum wind gusts are negatively correlated with several gases, such as carbon dioxide-MG811 (R = −0.8) and humidity (R = −0.51), indicating that high winds disperse some pollutants, lowering concentrations at the measurement locations. Temperature also impacts emissions, with minimum temperature (Tmin) showing a strong inverse correlation with CO_2_ (MG811: R = −0.55), suggesting a possibility of higher CO_2_ accumulation during cooler periods. Barometric pressure (MSLP) notably influences gas levels, with strong positive correlations across the H_2_S, NH_3_, C_6_H_6,_ and CO_2_ sensors (R = 0.55 to 0.82). Environmental humidity and LAI were unrelated (~90°) in PCA plots and the correlation matrix. The CTD and Ig remain directly related and inverse to TWSI as the drought conditions signal for the closed stomata and diminished photosynthesis. This analysis highlights the complex relationship between environmental factors and gaseous emissions, demonstrating that local weather patterns and plant physiology influence air pollution and vice versa. These insights are critical for air monitoring, automated irrigation, pollution mitigation strategies, and designing future urban GI.

### 3.5. Modelling Using ANN

**Figure 10:**
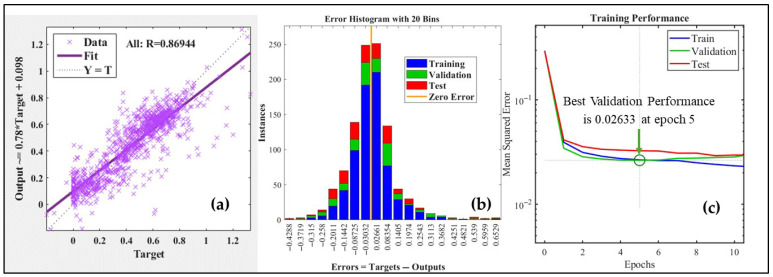
Artificial Neural Network training results, including (**a**) regression fit showing an R-value of 0.86944, (**b**) error histogram (errors as observed minus estimated) showing the normal distribution trend, and (**c**) Training performance of ANN in terms of mean squared error, with best validation performance demonstrated at epoch 5.

The ANN model designed to predict the logistically and computationally expensive variables (TWSI, Ig, CTD, and LAI), using the cost-effective E-nose sensors data, was trained in 11 iterations. The trained model had an MSE of 0.0249, 0.0242, and 0.0228, and an R-value of 0.8240, 0.8126, and 0.8335 for training, validation, and test datasets. Some unfiltered data was spared for the additional test and was not part of the training process. The trained model was run on this data, consisting of 258 observations, and yielded an overall 0.8694 R-value with a low MSE of 0.273. This approach ensured that the network could generalize across different trees, though from the same site. The performance of the network at different stages of training is statistically depicted in Table 4. The regression fit of this data, along with an error histogram, is shown in Figure 10a,b. The ANN prediction model minimizes the reliance on expensive equipment by leveraging affordable sensor data (input) to estimate crucial physiological parameters of tree canopies (output). With its high predictive accuracy of over 0.8694 (R > 0.8694) and low error rate of less than 0.3 (MSE < 0.273), this model offers a cost-effective solution for large-scale, real-time environmental and GI monitoring. The training performance of the ANN is depicted in Figure 10c, which shows a close agreement between the training and validation set mean squared error (MSE). This indicates effective convergence without signs of overfitting.

The different patterns of the studied parameters, as shown in bar charts and statistical analysis, demonstrate the modelling potential to use cost-effective data acquisition (E-nose and BoM) to predict experimentally and computationally expensive variables (LAI, TWSI, Ig, CTD). The ANN developed, as a result, manifests the proposition made earlier in this paper. The discernible grouping of spatial and diurnal data in our study, where atmospheric air pollutants have been monitored in conjunction with previously administered GI growth status, opens new horizons for applying deep learning algorithms on the collective data for selective irrigation and smart scheduling, not only to maximize canopy development but also to reduce the air pollutants.

### 3.6. Limitations and Future Work

While the measurements presented in this study were limited to one avenue (Royal Parade, Melbourne), the methodology and prototype are generally applicable to all types of urban GI. The incorporated methodology of GPS and timestamp linking with E-nose data, weather data from BoM, and thermal/visible image data is robust. Based on a new test site, the model would need to be retrained at least once. Also, the weather station (BoM) was located 3.4 km from the measurement site, which introduces small spatial uncertainties. The humidity/temperature values from sensors were compared to those acquired from BoM to ensure consistency.

The traffic count data was not acquired or incorporated in this study, but it could be a very valuable indicator. This parameter would be extensively investigated in future studies to provide better interpretations for variations in gas concentrations, particularly from vehicular emissions. The E-nose is located a few metres away from the tree canopy due to the height difference with the vehicle, limiting gas sensor readings to a height lower than the canopy. A future goal is to further enhance the deployment of the system by alleviating costs and operational complexity.

## 4. Conclusions

In the presented study, plant indices, environmental gaseous emissions, and meteorological parameters are jointly studied to mitigate solutions for effective irrigation, urban GI plantation, and pollution management. While infrared (IR) imaging systems are effective for large-scale environmental visualization, their limited sensitivity to non-IR-active gases limits their wide field deployment applications. Statistical evaluation revealed strong correlations between gas sensor outputs and key GI indicators (LAI, LAIe, TWSI, Ig), confirming the predictability of vegetation indices through environmental data. The findings of this study indicate that smart irrigation in urban green infrastructure can be efficiently managed using a cost-effective E-nose and computationally light ANN model. This study is the first to apply predictive modelling to estimate CTD, TWSI, Ig, and LAIe (calculated using thermal/visible cameras and computer vision algorithms) using the gaseous emissions measured via a compact, portable, and real-time E-nose. It is noticed that weather (sunlight, wind, pressure, ambient humidity) also has a complex relationship with the plant indices and air contaminants (VOCs). The proposed study has significantly simplified such complicated processes wherein the trained models can be integrated on top of a moving surveillance vehicle or public transport (buses/trams) with real-time data acquisition and decision-making for pollution control and optimized irrigation in urban GI. If commercialized and implemented, this study would revolutionize the contemporary methods of roadside GI.

## Figures and Tables

**Table 1:** Gas sensor modules, target analytes, and their MDLs for E-nose.

Sensor Module	Prime Specificity	Method Detection Limits	Other Detectable Analytes ^1^
MQ-3	Ethanol (C_2_H_6_O)	0.05 mg L^−1^–10 mg L^−1^	CO, LPG, C_6_H_6_, Hexanes, CH_4_, Alcohols
MQ-4	Methane (CH_4_)	200–10,000 ppm	CH_4_, LPG, H_2_, CO, Alcohol, smoke
MQ-7	Carbon monoxide (CO)	20–2000 ppm	CO, H_2_, LPG, CH_4_, Alcohol
MQ-8	Hydrogen (H_2_)	100–10,000 ppm	H_2_, LGP, CH_4_, CO, Alcohol
MQ135	Ammonia, alcohol, benzene	10–300 ppm, 10–300 ppm, 10–1000 ppm	Acentone, Toluene, NH_3_, Alcohol, CO, CO_2_
MQ136	Hydrogen sulphide (H_2_S)	1–100 ppm	H_2_S, NH_3_, CO
MQ137	Ammonia (NH_3_)	5–200 ppm	NH_3_, C_2_H_6_O, CO
MQ138	Benzene, alcohol, ammonia	10–1000 ppm, 10–1000 ppm, 10–3000 ppm	Benzene, n-Hexane, CH_4_, CO, Alcohol, Propane
MG811	Carbon dioxide (CO_2_)	350–10,000 ppm	CO_2_, C_2_H_6_O, CO, CH_4_

^1^ CO: Carbon Monoxide, LPG: Liquefied Petroleum Gas, C_6_H_6_: Benzene, CH_4_: Methane, H_2_: Hydrogen, CO_2_: Carbon Dioxide, NH_3_: Ammonia, H_2_S: Hydrogen Sulphide, C_2_H_6_O: Ethanol.

**Table 2:** Weather data collected from the Bureau of Meteorology, Melbourne, for all test days.

Weather Data (Bureau of Meteorology)																
Date	T_min_ (°C) ^1^	T_max_ (°C)	R (mm)	W.D	W.S (km h^−1^)	W.T_MAX_	T_9A.M._ (°C)	R.H._9A.M._ (%)	W.D_9A.M._	W.S_9A.M._ (km h^−1^)	MSL_9A.M._ (hPa)	T_3P.M._ (°C)	R.H._3P.M._ (%)	W.D_3P.M._	W.S_3P.M._ (km h^−1^)	MSL_3P.M._ (hPa)
19 April	12	20.6	0	NW	30	13:27	15.3	64	N	9	1018.4	18.9	47	NNW	15	1014.1
21 April	7.1	14.9	10.4	N	19	23:00	10	65	NW	2	1019.2	14.2	48	S	7	1015.4
22 April	9.9	17	0	WSW	30	16:15	13.4	62	NNW	13	1013.3	16.8	46	WNW	9	1012
29 April	8.2	18.4	0	SSW	17	13:44	12	83		Calm	1025.1	17.5	66	SSW	9	1022.9

^1^ T_min_: Minimum temperature, T_max_: Maximum temperature, R: Rainfall, W.D: Direction of wind, W.S: Speed of maximum wind, W.T_MAX_: Time of maximum wind, T_9A.M._: 9 a.m. Temperature, R.H._9A.M._: 9 a.m. relative humidity, W.D_9A.M._: 9 a.m. wind direction, W.S_9A.M._: 9 a.m. wind speed, MSL_9A.M._: 9 a.m. MSL pressure, T_3P.M._: 3 p.m. Temperature, R.H._3P.M._: 3 p.m. relative humidity, W.D_3P.M._: 3 p.m. wind direction, W.S_3P.M._: 3 p.m. wind speed and MSL_3P.M._: 3 p.m. mean sea-level pressure.

**Table 3:** Examples of growth and water stress metrics derived from measurement surveys conducted in April 2021 at Royal Parade (Melbourne, Australia) using the suggested urban tree monitoring system. The parameters for the leaf area index (LAI), effective LAI (LAIe), tree canopy temperature (Tc), canopy temperature depression (CTD), thermal infrared index (Ig), and tree water stress index (TWSI) are shown along with their minimum, maximum, mean, and standard deviation (SD) values.

	19 April 2021 (03:02:07 p.m.)				22 April 2021 (04:34:32 p.m.)			
	Min	Max	Mean	SD	Min	Max	Mean	SD
**LAI**	1.3 × 10^−5^	4.147	0.925	0.968	0.0005	3.191	0.988	0.799
**LAIe**	1 × 10^−5^	3.690	0.763	0.802	0.0005	3.105	0.966	0.781
**Tc**	11.784	25.568	22.888	1.765	3.473	18.326	10.566	3.565
**CTD**	−7.316	6.668	3.682	1.783	−11.127	4.326	−3.827	3.652
**Ig**	0.208	1.235	0.626	0.213	0.229	1.292	0.568	0.252
**CWSI**	0.447	0.828	0.625	0.077	0.436	0.813	0.653	0.095

**Table 4:** Statistical results for the artificial neural networks fitting tool model for estimation of (TWSI, Ig, CTD, and LAI) using the E-nose sensor values (MQ-3, MQ-4, MQ-8, MQ-135, MQ-136, MQ-137, MQ-138, MG811, Temperature, Humidity).

Stage	Observations	MSE	R
Training	353	0.0249	0.8240
Validation	76	0.0242	0.8126
Test	76	0.0228	0.8335
Additional Test	258	0.0273	0.86944

## Data Availability

The original contributions presented in this study are included in the article. Further inquiries can be directed to the corresponding author.
